# Emirati Adolescents’ and Young Adults’ Usage of Social Media for Health Information

**DOI:** 10.3390/children10101636

**Published:** 2023-09-30

**Authors:** Hiba Jawdat Barqawi, Kamel A. Samara, Hanae Qousae Al Chame, Ibrahim Marouf Al Shyyab, Mariam A. Almaazmi

**Affiliations:** Department of Clinical Sciences, College of Medicine, University of Sharjah, Sharjah 27272, United Arab Emirates; u17103231@sharjah.ac.ae (K.A.S.); u18100478@sharjah.ac.ae (H.Q.A.C.); u17100161@sharjah.ac.ae (I.M.A.S.); u17100724@sharjah.ac.ae (M.A.A.)

**Keywords:** adolescents and young adults, social media, health information, internet, professionalism, eHealth

## Abstract

During the COVID-19 pandemic and in the years after, adolescents’ and young adults’ (AYAs) usage of social media increased. Social media has been shown to influence both the physical and mental behaviour of AYAs. The Emirates’ AYAs are among the world’s heaviest consumers of social media. This study aims to explore the usage of social media networks by AYAs for sharing and looking up health information, as well as interacting with local health systems, with a special focus on doctors and social media. This cross-sectional, descriptive study was used to collect comprehensive data from Arabic- and English-speaking Emirati students in grades 7 through 12 and university students between the months of December 2022 and April 2023. A total of 876 out of 930 responses were included. Of the responses, 27.71% were university students, with another 46.46% in grades 11 and 12. Three-fourths had a hospital or clinic visit in the last 6 months and 79.11% had good health perception. Smartphones were the most commonly used devices, at 92.12%. A total of 74.7% reported being able to obtain useful health information, with 40% having had a health decision influenced by it. Posting information on social media was not common, with only 32% posting such content (most commonly mood-related). Health information on social media by health authorities was considered the most trustworthy, with celebrities being the least trustworthy. More than half of AYAs searched for a physician before a visit, and the majority would not mind having a rash picture being posted on a public website if consent (verbal or written) was taken. Social media can influence the various health decisions an AYA might take and which doctors they might choose to see. Physicians should consider their presence and the content they present on social media carefully.

## 1. Introduction

Social media refers to “Internet-based tools that allow individuals and communities to gather and communicate; to share information, ideas, personal messages, images and other content” [1]. Examples of social media include wikis, networking sites (such as Facebook and LinkedIn), media sharing sites (such as YouTube and Instagram), and blog sites (such as Twitter) [2]. In 2022, 97% of American teens reported using the internet daily, with 46% stating continuous usage. At 95%, YouTube was the most commonly used platform, followed by TikTok at 67%, and Instagram at 62%. Indicative of the highly dynamic nature of these networks, Facebook, a previously dominant network, ranked 5th, with only 32% reporting using the platform, which dropped from 71% back in 2014–2015 [3]. Follow-up work by the Pew Research Center also discovered that, in the following years, during the COVID-19 pandemic, parents reported an increase in their children’s usage of electronic devices and social media, coupled with an increase in parental concerns [4].

Social media has been shown to influence both the physical and mental behaviour of adolescents and young adults [5,6,7]. Additionally, future patterns of adult health are established during childhood and adolescence and may continue into old age [5]. This can lead to social media networks being used for health intervention and promotion. Hunter et al. has shown that these interventions are effective in both the short and long term for health outcomes, including well-being and smoking cessation [8]. Health benefits of social media can include enhanced access to valuable support networks, which are particularly helpful for patients with ongoing illnesses, conditions, or disabilities [9]. Patients can benefit from social media by connecting with others with similar conditions, joining support groups, or researching health information. In an urban study in the United States, most participants were primarily looking to connect with others and receive advice and support [10]. For example, Facebook enables patients to aggregate and form their own groups, most of which are centred around one of four themes: fundraising, awareness, marketing, and general support [11].

However, there are a platitude of concerns regarding the use of social media for health by AYAs. Information on social media could be from regulated sources (such as governments or health organisations) or unregulated sources (such as peers and celebrities) [12]. Even more worrying is that a sizable majority may not recognise that they are actually using these technologies for health information. In a study among 13–18-year-olds, only 3.5% reported using their phones for health information, but up to 91.7% reported health information seeking [7]. Moreover, and considering the COVID-19 pandemic had huge effects on university activities such as teaching and research, those AYAs will grow into a world where the internet and social media have become ingrained in professional activities [13].

Studies that have looked at online health content have found worryingly high amounts of content with misleading and downright harmful content, all of which is on an upward trajectory [14,15]. Moreover, AYAs have not been shown to recognize reliable and trustworthy online health information sources. Most participants in Plaisime et al’s study. could not identify two reliable sources and used unreliable indicators to gauge the trustworthiness [7]. Hence, while some adolescents and young adults may be critical users of social media for health information, results have shown that a portion are vulnerable and influenced to act on health-related information that is potentially harmful to them [12]. However, recent work has begun empirically evaluating the issue of media manipulation from the point of view of young adults, outlining both a theoretical framework and practical and addressable risk factors that predict higher rates of manipulation success [16].

Countries of the gulf region, including the United Arab Emirates (UAE) and Saudi Arabia, are among the world’s heaviest consumers of social media. Yet, the level of research exploring social media and health promotion in the region is insufficient, especially locally, in the UAE. In fact, in a review by AlSadrah, out of a total of 30 studies exploring social media and health, none was focused on the UAE [17]; this is surprising given the massive consumption of social media seen in the country. However, over the last few years, more research has begun to be conducted in this area: Hegazi et al. developed a social media-based instrument to evaluate mental health stigma in the Emirates [18], while Al Kazhali et al. have explored the links between social media and poor sleep quality [19]. As such, this study aims to set the stage for further research by reporting on the usage of social media networks by adolescents and young adults for sharing and looking up health information, as well as interacting with local health systems.

## 2. Methodology

### 2.1. Study Population

This cross-sectional, descriptive study was used to collect comprehensive data from the UAE. Emirati students in grades 7 through 12, along with any university students, were included. Data collection was conducted between the months of December 2022 and April 2023 using convenience sampling through WhatsApp groups and other social media platforms, such as Twitter and Instagram. The minimum required sample size was found to be 385 participants, using Cochran’s sample size formula using a confidence level of 95%, a sampling error of 5%, and a standard error of 1.96. A total of 930 responses were collected, out of which only 876 were retained after removing those who did not meet the inclusion criteria. A participant information sheet (PIS) was presented before starting the questionnaire, and filling in the questionnaire indicated consent to participate in the study. Finally, the collected data was available only to the investigators to ensure confidentiality, and no identifying data was collected.

### 2.2. Questionnaire Development

The tool used for exploring social media usage for health information by AYAs was adapted from the one used by [10]. The questionnaire was originally developed in English and then translated to Arabic. The Arabic version was reviewed multiple times to ensure consistency with the original. Both were pilot tested two times; all the provided feedback was evaluated and incorporated if appropriate. The 37-item self-administered questionnaire consisted of three different sections: demographics, social media usage and views, and social media interaction, trustworthiness, and engagement. It included 5-item Likert scales, as well as true and false and single-select/multi-select questions. This research was reviewed and approved by the Research Ethics Committee of the University of Sharjah (Reference Number: REC-21-04-29-01-S) on 29 April 2021. It was conducted in accordance with all relevant guidelines and regulations.

### 2.3. Statistical Analysis

Data was exported from Google Forms to CSV format and processed in python-3 using the Matplotlib-v3.3.4, pandas-v1.2.4, and statsmodels-v0.12.2 packages for analysis and interpretation. All cases with missing values or inconsistent answers were dropped. Frequency distributions were calculated for categorical variables. The 5-item Likert scales were collapsed to binary variables, combining responses of 4 and 5 to above average and the rest to average or below.

## 3. Results

### 3.1. Demographics of Participants

A total of 876 responses from Emirati students were included in this study. Of the responses, three-quarters were male, with nearly half being high school students. A quarter did not have any visits to a clinic or hospital in the last 6 months, and 79.1% rated their health as above average. The sample was nearly equally distributed among the three major Emirates groups, with Abu Dhabi being most represented at 40.1%. With regards to devices being used, mobile phones were by far the most common at 92.1%, followed by laptops at 35.1%. The full demographics can be seen in Table 1.

### 3.2. AYA Social Media Interaction

A total of 74.7% reported that social media can help them obtain useful health information. Interestingly however, more than half did not consider social media as a main source of any health-related information. A third reported using it as a main source for wellness and prevention health information (such as that related to sleep or diet), making this the most common use. Similarly, 68% did not post any health-related information on their social media. The most common type of information posted was related to mood at 16.4%, followed by wellness and prevention at 14.8%. Snapchat was the most common platform at 16.8%, followed closely by Instagram at 14.2%. Only 5.9% shared health information on WhatsApp, and nearly 1% posted on Facebook or dedicated health blogs.

**Figure 1:**
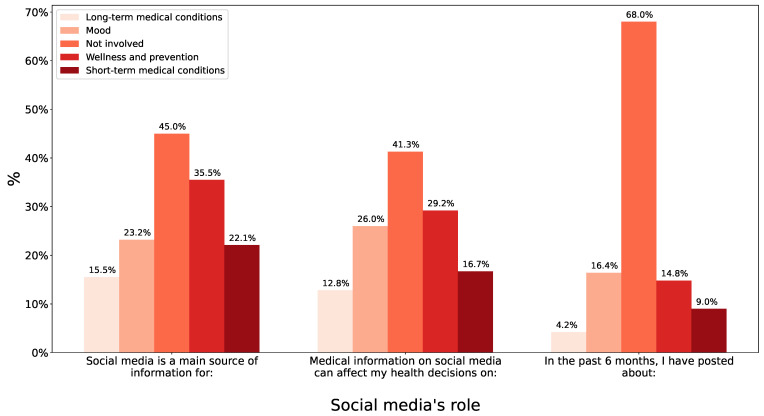
Social media’s role in various health-related information practices, including being a main source of knowledge for that type of health information, influencing health decisions, and being a frequent target to post health information.

When it came to posting health information on social media, reasons varied greatly. The most common was to seek advice, at nearly 10%. However, the other reasons were also close, with percentages ranging from 5 to 8%, the reasons being connecting with others with similar conditions, sharing the health issue, searching for additional treatment options, and looking for support. Yet, nearly 40% of the participants had their health decisions influenced by social media, again with prevention being most common at 29.2%, followed by mood at 26.0%. Figure 1 displays social media’s role as a source of, affecting, and posts related to the various health information asked about.

However, not all health information on social media was treated equally. The most trusted information was that being shared by health authorities, with 83.7% ranking it as being highly or very highly trustworthy. Yet, interestingly, 44.0% did not follow any health authority on social media. The most common health authority being followed was the UAE’s Ministry of Health and Prevention (MOHAP) at 31.9%, followed by nearly 22% for each of Abu Dhabi’s Department of Health, Dubai’s Health Authority, and the World Health Organization. Hospitals and doctors garnered similar trustworthiness levels at 80.9% and 77.1%, respectively. Family and friends came next at 61.1%. Celebrities were considered the least trustworthy, with only 20.3% considering health information coming from them being highly or very highly trustworthy.

### 3.3. Doctor and Social Media Usage

**Figure 2:**
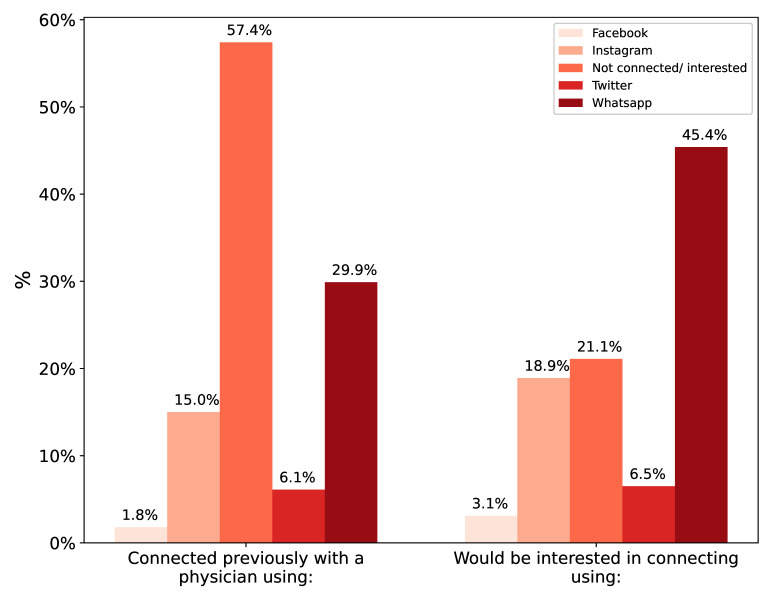
Methods for connecting with physicians.

Given the high trustworthiness of doctors on social media and the multitude of such accounts, further exploration of doctors and social media was conducted. First, the majority (57.4%) of participants had never connected with a physician treating them on any social media platform. The most common platform for connecting was WhatsApp at 29.9%, followed by Instagram at 15.0%. Yet, there is overwhelming interest in connecting with physicians, with only 21% stating otherwise, most commonly through WhatsApp (45.4%), followed by an interactive clinic website (31.6%). Figure 2 shows the connection rates for other commonly used social media platforms. The most common reasons for using social media to contact physicians was “easier to communicate” at 48.9%, followed by “more responsive doctor” at 27.6%. Only 21.0% reported that they would feel more connected to the doctor through social media communication.

However, physicians’ social media profiles can also be used for a variety of other reasons in addition to direct communication or health information promotion. Of the surveyed participants, 56.9% reported searching for a doctor on social media before a visit. Reasons for this were split nearly equally among three main reasons: checking how long a physician has been working (27.7%), reading any reviews or comments (26.3%), and checking qualifications (25.5%). Only 10.6% were checking because they were curious about a doctor’s appearance. However, some physician personal content on social media can also influence patients’ views and attitudes. Only 15% reported a decrease in trust if they saw personal photographs of a doctor (such as on vacation or with family and friends), with 69.4% reporting no change in trust. However, with inappropriate posts or comments, disrespectful stories about patients (without exposing identity), and posts that potentially reveal patient identities, nearly 60% reported a decrease in trust for each situation. Interestingly, respectful stories about patients (without compromising patients’ identities) caused an increase in trust in nearly half the participants, with more than a third reporting no change.

**Figure 3:**
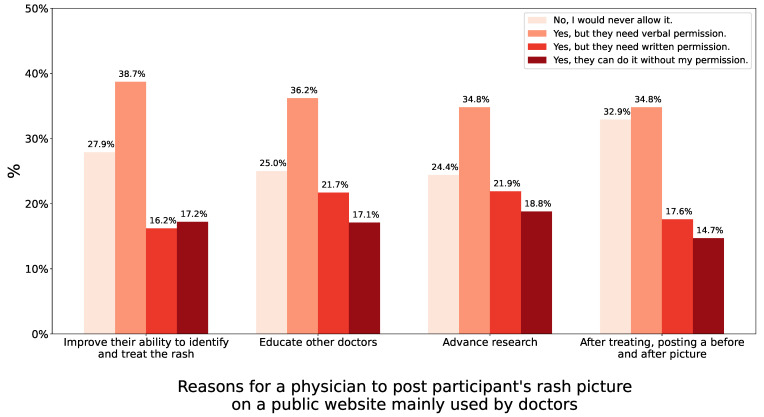
Rash case study.

Finally, participants were presented with a scenario where they had a rash on their back that a physician was treating. They were asked if they would allow the doctor to post a picture of the rash, for various reasons, on a website used mostly by. The reasons included helping the physician recognize and treat the rash, educating other physicians, advancing research, and highlighting before and after treatment response. Overall, more than two-thirds would support such an action. However, the majority again favoured the doctor asking for some permission, verbal being more common than written. Figure 3 shows the various responses to the possible reasons.

## 4. Discussion

In this study, the aim was to study the usage of social media among Emirati adolescents and young adults for health information, as well as explore the effect on their practices and views. It was found that the usage of social media for health was high, with nearly three-quarters obtaining health information from social media. Prevention-related information was both the most searched for on and most influenced by social media. Sharing of health information was less frequent, with two-thirds not posting any material regarding their health. Trust of information on social media also varied, with health authorities being the most trusted, with an 83.7% highly/very highly trustworthy rating. The most common platform for connecting with physicians was WhatsApp, followed by Instagram; similarly, there was widespread interest for connecting with physicians through social media networks. Trust in physicians was highly affected by negative posts. Finally, the majority of participants would consent to a back rash being posted on social media, especially if permission was taken from the patient.

In 2023, nearly 99% of the UAE’s population was active on social media, with an average daily time of 2 h and 50 min. YouTube is the most common platform, at 79.60%, followed by Facebook, Instagram, and TikTok at 78.70%, 73.40%, and 67.40%, respectively, while WhatsApp is the most common chat platform, at 80.2% [20]. Additionally, in the Middle East, users have been found to frequently share and exchange sensitive health information online, especially when it comes to sensitive topics such as HIV or depression. In one study, 31% of the Facebook posts reviewed involved sharing experiences, followed by explicit queries or advice offering at 13.6% and 8.3%. The study highlighted a lack of laws and legislations that protect and empower patients against misuse of their sensitive information shared through social media [21]. In this study, a high usage of social media was seen, but the participants were much less likely to share information regarding their health. These results are also in line with a previous study that looked at youth and their engagement with social media locally and found 81% of the participants regularly used social media for health information, with WhatsApp being the most frequently utilised tool [22].

Additionally, web-based social health networks help patients expand their knowledge, which can lead to better patient involvement and patient activation; this ultimately manifests through higher levels of self-efficacy and better knowledge and behaviours [23]. Young adults describe the benefits of seeking health information online and through social media and recognize these channels as useful supplementary sources of information to health care visits [2,24]. Patients use social media as a way to supplement rather than circumvent their doctors [11,25]. Yet, in this study, more than half of the participants did not recognize social media as a health information source even though the overwhelming majority used it and more than a third had their decisions influenced by it.

Social media, however, poses a number of risks and barriers that need recognition. In a study of health indicators among adolescents in the UAE from 2005 to 2016, Pengpid and Peltzer found a rise in the rates of bullying, overweight individuals and obesity, sedentary behaviours, and lack of physical activity [26]. All of these can also be exacerbated with inordinate consumption of social media, which the local youth have been found to engage in [27]. A large Dubai study including 7000 students across 10 schools similarly found a high usage of electronic devices among AYAs, with nearly 70% spending between an hour and five hours every day on social media. Additionally, a number of burdens and negative impacts of social media were reported, with 55% stating it interfered with homework and another 60% expressing how it reduced their interaction with the outside environment and family time [28].

Moreover, with the use of platforms, there is a major concern regarding the users’ confidentiality and privacy [29]. AYAs may not be able to fully gauge the impact of sharing health information on such applications, and that can open them up to exploitation. Moreover, the sharing or discussion of health information can also expose them to trolling (deliberate provocation) or flaming (mocking or encouraging self-harm), leading to increased suicidality [2].

### Doctors and Social Media

The massive explosion of social media has led to the blurring of personal and professional lives for some, with doctors being no exception. This heralded the rise of e-professionalism, also known as online or digital professionalism by Cain and Romanelli and defined as “attitudes and behaviours … reflecting traditional professional paradigms manifested through social media” [30].

Globally, it has been found that physicians are avid users of social media. A review by González-Teruel et al. on medical residents found that the majority reported having social media accounts. Interestingly, it was found that most residents were passive users of social media, yet most had a preference for personal use over professional. Moreover, the review found that in 13 studies that evaluated professionalism, all found clear incidents of professional violations, though infrequent. Examples include alcohol consumption or images identifying patients [31]. A study among Saudi family physicians and residents found that nearly all participants had at least one social media account, with the median time spent being 120 min. Moreover, 75% reported using them for personal reasons, with YouTube being most commonly reported at 74%, followed by Twitter at 58% and Facebook at 48% [32]. The usage among Saudi physicians was actually much higher compared to other global studies, where healthcare practitioners averaged usage patterns between 50% and 80% [33].

In this study, it was found that there is substantial demand for connecting with physicians over social media, aligning well with the results from neighbouring Saudi Arabia. However, it can also be argued that the wide usage of social media is not driven by the demand of the patients but by the desire of the providers. As such, this can also lead to the sharing of inappropriate material by the physicians. A review by Guraya et al. looking at social media usage among HCPs found a rise in unprofessional behaviours on social media along with a clear absence of social media policies, threatening patients’ confidentiality and physicians’ professionalism and integrity [34].

In a local study, while nearly 90% of pharmacy and medical students believed it would be discriminatory to use social media material for hiring decisions, two-thirds admitted to sharing material they would not want their employers to see [35]. In fact, social media can damage HCPs’ trustworthiness, either by providing poor-quality information or damaging the professional image. This is coupled with differences in perceptions of inappropriate social media behaviour, especially among the younger generations. For instance, some medical students regarded alcohol or dress themes as online social identity rather than unprofessional behaviour, in contrast with professional bodies [33]. In this study, it was clear that AYAs are much more likely to be negatively influenced by social media mishaps (such as inappropriate posts or stories) than positively influenced. This can damage the high level of trustworthiness in doctors and health authorities that was seen in this study and many before [36,37] As such, it may be important for hospitals and clinics to develop social media guidelines that help doctors protect themselves and their patients.

## 5. Conclusions

With the ever-rising number and usage of social media networks, adolescents and young adults represent a population with unique and important challenges. Social media has been shown to influence both the physical and mental behaviour of adolescents and young adults, with studies also showing how it can be used for positive health intervention and promotion. Additionally, patients can benefit from social media by connecting with others with similar conditions, joining support groups, or researching health information. The United Arab Emirates is one of the world’s largest consumers of social media, yet there is a paucity of research on the topic. Therefore, this study aimed to study the usage of social media among Emirati adolescents and young adults for health information, as well as explore the effect on their practices and views.

Overall, this study has shown how social media has crept into and influenced the various health decisions an AYA might make and which doctors they might choose to see. Additionally, and knowing how social media has led to the blurring of personal and professional lives for some, physicians should consider their presence and the content they present on social media carefully. Similar to the general population, doctors have also been found to be avid consumers of social media, and this study has shown how content shared can shape patients’ views (including AYAs) and negatively influence and affect the patient–physician dynamic that is essential to any healthcare interaction. As such, the results highlight the importance for hospitals and clinics to develop social media guidelines that help doctors protect themselves and their patients.

## Figures and Tables

**Table 1:** Demographics.

Feature	*n* (%)
**Sex**	
Female	223 (25.46%)
Male	653 (74.54%)
**Educational level**	
School (grades 7 to 10)	231 (26.37%)
School (grades 11/12)	407 (46.46%)
College/university	238 (27.17%)
**Visits to any clinic or hospital in last 6 months**	
None	221 (25.23%)
1	224 (25.57%)
2–4	311 (35.5%)
5+	120 (13.7%)
**Health Perception**	
Average or below	183 (20.89%)
Above average	693 (79.11%)
**Devices used**	
Smartphone	807 (92.12%)
Laptop	307 (35.05%)
Tablet	193 (22.03%)
**Emirate (Residence)**	
Abu Dhabi	351 (40.07%)
Dubai	209 (23.86%)
Sharjah and other Northern Emirates	316 (36.07%)

## Data Availability

The datasets used and/or analysed during the current study are available from the corresponding author on reasonable request.
